# Impact of technological innovation and regulation development on e-waste toxicity: a case study of waste mobile phones

**DOI:** 10.1038/s41598-018-25400-0

**Published:** 2018-05-08

**Authors:** Yu Chen, Mengjun Chen, Yungui Li, Bin Wang, Shu Chen, Zhonghui Xu

**Affiliations:** Key Laboratory of Solid Waste Treatment and Resource Recycle (SWUST), Ministry of Education, Southwest University of Science and Technology, 59 Qinglong Road, Mianyang, 621010 China

## Abstract

Technology innovation has accelerated progress in Information and Communications Technology (ICT), especially in the mobile phones sector. Concurrently, local, national, and international governments are enforcing stricter regulations to protect natural resources and human health. The paper attempts to address the question: Have technological innovations and regulation development had a positive impact on ecosystems and public health? We identified 36 waste mobile phones (WMPs) manufactured between 2002 and 2013, assessed their metals concentration, leachability, and potential impact on environment and human health using digestion, Toxicity Characteristic Leaching Procedure (TCLP), and USEtox model, respectively. The results highlight that regulations did not have significant impact on total metal content, except some heavy metals, while technology innovation recorded stronger impact. WMPs should be classified as hazardous due to excessive lead content. Copper posed the most significant ecotoxicity risk, and chromium showed the most significant risk for both cancerous and non-cancerous diseases. Additionally, we demonstrated that WMPs toxicity increased with technology innovation.

## Introduction

The first mobile phone invented by Marty Cooper 44 years ago, weighed 2.5 pounds, was 9 inches in length, 5 inches in thickness, required ten hours to charge, and functioned for 20 minutes^1^. Today, mobile phones are versatile and work like professional computers and cameras; are much lighter, compact, beautiful, and intelligent; and have become an indispensable part of human lives^2^. Accelerated innovation has lead to proliferation in mobile phone production. International Telecommunication Unions^3^ reported that 781 million mobile phones were generated in 2015 and the numbers will increase to 877 million units by 2020. However, rapid innovation has also reduced the usage span of phones^4^, resulting in increase in the number of waste mobile phones (WMPs), categorised as waste electric and electronic equipment (WEEE), also called as electronic waste or e-waste^5,6^. WMPs and waste printed circuit boards (WPCBs) are listed as one of hazardous wastes by the U.S., European Union, China, and other nations^7–9^.

E-waste is the core of “urban mining” due to abundant content of secondary materials, especially valuable metals such as copper, gold, and palladium^10,11^. Gold contained in WMPs are higher than other e-waste. For example, gold in WPCBs of WMPs is 300 g per ton compared to 100 g per ton found in WPCBs of desk computers^12^. Consequently, WMPs can be considered as the core of e-waste. At present, recovery, reuse, and recycling are considered as the most effective approaches to WMPs management^13^. However, only 10% of the end-of-life mobile phones are recycled in the U.S.; the residual 90% are stored at homes by users or are dumped in landfills^14^, where they leach toxic substances into the environment and threaten the ecosystem and human health^15,16^.

Toxic substances including heavy metals such as lead, zinc, chromium, cadmium, and brominate flame retardants like PBBs and PBDEs threaten the ecosystem and human health, especially when treated improperly^17,18^. Although regulations vary across countries, they are increasingly stricter due to environmental and public health concerns^19^. In the past 20 years, local, national, and international governments have enacted series of regulations and laws to restrict the use of hazardous materials in information and communication equipment^20^. The best examples are the “Directive on the restriction of the use of certain hazardous substance in electrical and electronic equipment” (RoHS) and the “Waste Electrical and Electronic Equipment Directive” (WEEE) by the European Union which specify the thresholds for six hazardous substances. Meanwhile, electric and electronic equipment (EEE) industry pursuits have persisted on technology innovation by applying new materials and restricting hazardous substances in response to public awareness of environmental protection and cost reduction^7^. Innovation is especially significant in the Information and Communication Technology (ICT) industry and the mobile phones sector. Consequently, this research addresses the question: “Will such significant changes in materials resources caused by regulation development and technology innovation reduce the chemical toxicity risk of WMPs?” To the best of our knowledge, this issue has not been previously investigated.

We collected WMPs generated between 2002 and 2013 and analyzed metals to assess the effect of technological innovation and regulations. We conducted chemical leaching assessment procedures to evaluate if the WMPs should be classified as hazardous waste. We also employed a life cycle impact model, USEtox^21,22^, to evaluate the ecological toxicity and human health (cancer and non-cancer related) impacts of WMPs caused by technological innovation and regulations. We expect these results will provide valuable information to guide the administration and industry to set up cost-effective and efficient approaches to eliminate chemical toxicity risks of electric and electronic products.

## Materials and Methods

### Sample preparation

In 2014, we conducted internet searches and identified nearly 1000 mobile phones models produced from 2000 to 2013. We chose one mobile model produced by the top three or four manufacturers each year. The top manufacturers were mainly NOKIA, SAMSUNG, MOTOROLA, BLACKBERRY, and APPLE^23,24^. Thus, we identified 52 mobile phones manufactured between 2000 and 2013, which are listed in Supporting Information (SI) - Table 1. Subsequently, we searched the market and several mobile phone recycling companies to collect all identified WMPs. We collected 36 WMPs over one and a half years, though some were broken, short of battery, or lacked back shells. Detailed information about the 36 cellular phones is given in SI Table 2. We classified WMPs into two categories: Group 1- without any physical parts missing; and Group 2 - without battery or back shell, as detailed in SI Table 2.

All WMPs were weighed, disassembled, and crushed using a mill (SM-2000, Retsch, Germany) to particles of diameter around 9.5 mm for TCLP (Toxicity Characteristic Leaching Procedure, U.S.E.P.A. 1992) testing. The obtained powder samples were stored in marked airtight polyethylene bags for further analysis.

### Metal analysis and hazardous test

HF-HClO_4_-HNO_3_ system was used to digest the powder samples, as described elsewhere^25^. The metals in the digested solutions were analyzed using inductively coupled plasma-optical emission spectrometer (ICP-OES, Perkin Elmer, Optima 8300, USA). In this research, 22 elements namely aluminium, arsenic, antimony, barium, beryllium, cadmium, chromium, cobalt, copper, gold, iron, magnesium, molybdenum, lead, nickel, palladium, selenium, silver, thallium, tin, vanadium, and zinc were selected.

The TCLP (Method 1311), which is designed to determine the mobility of chemical substances in liquid, solid, and multiphase wastes, is widely used in research to test potential hazard levels^9,26^. Six elements including arsenic, barium, cadmium, chromium, lead, and silver were tested in the WMPs using the TCLP.

### Life cycle impact assessment using USEtox

USEtox is a scientific environment model used to characterize potential impact of toxic chemicals in products on human toxicology and ecotoxicology. The model outputs the environmental fate, effect parameters, and also improves understanding and management of chemicals in the global environment by further applying the model to describe the exposure and effects of chemicals^27^. It was developed under the auspice of the United Nations Environment Program (UNEP) and the Society for Environmental Toxicology and Chemistry (SETAC). The researchers continue to update the model and factors of USEtox which updated from Version 1.01 (2010) to Version 2.02 (2016). In this study, we chose the USEtox “mid-point effect” characterization approach rather than the “end-point effect” in Version 2.02, which minimizes inference of data and uncertainties caused by interactions between different impacts^22^. The potential carcinogenic and non-carcinogenic impacts of human toxicity and eco-toxicity of the selected metals were calculated according to the formula:

1 $${P}_{x}={C}_{x}\cdot W\cdot W{f}_{x}$$

where, *P*_*x*_ represents the impact score of metal *x* in the WMP; *C*_*x*_ is the concentration of metal *x* in the WMP (kg/kg, Table 1); *W* is the total weight of the sample (kg, SI Table 2); and *Wf*_*x*_ is the characterization factor for the corresponding potential of metal *x*. The units of the characterization factor for human toxicity and ecotoxicity were cases/kg_emitted_, and PAF·m^3^·day·kg^−1^, respectively. The characterization factors derived from USEtox were associated with the impacts of metals emitted to household indoor air, industrial indoor air, urban air, rural air, fresh water, sea water, natural soil and agricultural soil.

**Table 1:** Metal content in waste mobile phones.

Year of production	Model	Ag	Al	As	Au	Ba	Be	Cd	Co	Cr	Cu	Fe	Mg	Mo	Ni	Pb	Pd	Sb	Se	Sn	Tl	V	Zn
2002	NOKIA 7650	725	28897	106	46	1502	N.D.	N.D.	15294	1896	23360	9944	1175	26	16416	1595	78	118	112	3457	N.D.	36	1433
2002	MOTO V70	384	33571	N.D.	54	4001	N.D.	N.D.	376	21459	20438	31689	1428	433	17248	2031	93	500	N.D.	6548	N.D.	45	3631
2003	NOKIA 1100	360	20553	50	N.D.	1051	N.D.	N.D.	311	4955	28934	24215	2184	44	3219	532	80	37	163	2472	N.D.	31	184
2004	NOKIA 7610	444	17633	N.D.	N.D.	2305	N.D.	N.D.	158	9799	24547	31823	2745	56	12985	271	85	106	43	2043	240	49	10063
2004	MOTO V3	487	28534	N.D.	N.D.	5814	N.D.	N.D.	1083	10276	20721	26356	12189	67	10614	45	64	1514	72	7347	N.D.	585	2187
2005	SAMSUNG D508	708	48764	117	53	711	N.D.	N.D.	9117	233	27883	3383	1915	8	29351	504	60	470	N.D.	2949	N.D.	42	162
2005	NOKIA N90	1110	31560	N.D.	N.D.	788	N.D.	N.D.	602	21749	28022	37649	7322	158	19799	69	60	471	N.D.	6043	20	135	45777
2005	SONY ERICSSON K750c	3160	23310	N.D.	N.D.	2005	N.D.	N.D.	203	20541	23510	32614	1210	97	11928	370	47	373	N.D.	6209	303	N.D.	1441
2006	NOKIA 5200	478	20420	N.D.	57	4224	N.D.	N.D.	573	12289	25364	33543	2744	196	10742	207	69	95	N.D.	10687	37	17	20473
2006	SAMSUNG SGH-D908	197	62363	46	N.D.	2415	N.D.	N.D.	15820	11690	37472	44486	19052	101	6927	33	83	120	12	3538	N.D.	265	492
2006	SONY ERICSSON W700C	458	12630	N.D.	N.D.	1369	N.D.	N.D.	682	14887	36902	49089	342	46	9820	90	64	244	N.D.	3350	26	54	1519
2006	MOTO A1200	182	11421	N.D.	N.D.	7797	N.D.	N.D.	384	10579	35233	43419	2551	25	20589	123	66	97	N.D.	900	135	36	172783
2007	IPHONE 1	347	44738	488	N.D.	707	N.D.	N.D.	947	56567	25960	44151	14878	506	27822	N.D.	34	875	N.D.	2565	129	267	1192
2007	NOKIA N95	154	24550	26	46	1207	N.D.	N.D.	174	16352	27467	36985	15177	75	16860	113	62	232	N.D.	4113	N.D.	942	107922
2008	NOKIA E71	445	7276	N.D.	N.D.	1525	N.D.	N.D.	792	77687	29300	48388	7058	580	54438	114	46	1162	N.D.	1671	46	260	29330
2008	BLACKBERRY 9000	588	17689	1023	2	2254	N.D.	N.D.	87	9226	33977	43306	2806	54	11855	115	103	88	88	5796	90	20	9356
2008	SAMSUNG i908E	283	47379	N.D.	N.D.	432	N.D.	N.D.	575	39310	33465	51697	737	452	33145	25	32	567	N.D.	1550	N.D.	204	2344
2008	IPHONE 3 G	123	24228	N.D.	N.D.	1840	N.D.	N.D.	29155	37680	24259	38064	8404	189	23355	47	71	489	N.D.	8362	N.D.	183	2287
2008	GOOGLE G1	1159	9783	N.D.	N.D.	1159	N.D.	N.D.	1168	42907	35028	52765	21289	458	22104	N.D.	64	805	N.D.	2934	N.D.	574	3757
2009	NOKIA N900	226	12965	N.D.	N.D.	596	N.D.	N.D.	1028	63286	27497	46179	2471	782	33488	51	35	864	N.D.	2235	67	196	4201
2009	SAMSUNG S5230	642	19759	N.D.	N.D.	1518	N.D.	N.D.	837	35496	25056	39462	16466	195	19044	N.D.	41	454	N.D.	7115	156	842	855
2009	IPHONE 3GS	95	23048	N.D.	N.D.	1970	N.D.	N.D.	31329	26678	33768	49007	5201	119	15876	N.D.	74	301	N.D.	7164	N.D.	76	1335
2010	SAMSUNG Galaxy S	483	29202	63	102	2533	N.D.	N.D.	322	8115	23392	31065	14814	172	6909	83	62	127	N.D.	7982	N.D.	757	1124
2010	IPHONE 4	106	26439	N.D.	N.D.	623	N.D.	N.D.	58599	27631	34067	46993	10759	378	15310	86	127	348	72	6008	N.D.	180	332
2011	BLACKBERRY 9900	206	11961	N.D.	N.D.	1170	N.D.	N.D.	748	58279	25255	40572	3514	312	33246	257	63	798	N.D.	6977	N.D.	167	578
2011	IPHONE 4 S	25	27152	N.D.	N.D.	475	N.D.	N.D.	40869	40536	22472	36264	7317	343	18990	19	91	540	N.D.	3568	N.D.	178	2387
2011	SAMSUNG Galaxy Note	4304	31538	138	69	1210	N.D.	N.D.	251	841	32903	21711	20855	62	3658	29	73	N.D.	73	4320	N.D.	972	4215
2011	GOOGLE Nexus S	1924	25989	85	73	999	N.D.	N.D.	47	2025	27102	33636	16423	54	4464	2011	57	N.D.	N.D.	10290	N.D.	829	26033
2012	SAMSUNG Galaxy Note II	302	27428	18	N.D.	3883	N.D.	N.D.	317	10917	29752	39366	19946	146	9473	99	70	127	N.D.	6127	107	686	2810
2012	IPHONE 5	114	59322	N.D.	N.D.	980	N.D.	N.D.	43279	22923	30072	43229	2559	74	15761	7	104	292	N.D.	4143	N.D.	222	101
2012	SAMSUNG galaxy nexus	388	27927	95	35	2041	N.D.	N.D.	550	271	29965	2552	19309	94	2225	18	55	N.D.	N.D.	2504	N.D.	889	1258
2012	BLACKBERRY 9850	257	12599	N.D.	N.D.	4284	N.D.	N.D.	330	22333	26871	38023	14566	88	14993	60	111	326	27	5327	26	235	151
2012	GOOGLE Nexus 4	364	28580	36	N.D.	2244	N.D.	N.D.	47462	11928	26301	33692	15859	103	10211	93	138	89	N.D.	8493	N.D.	470	2838
2013	SAMSUNG GalaxyNote3N9000	347	45281	153	20	15711	N.D.	N.D.	201	762	29997	5802	19481	116	11714	15	51	N.D.	46	4139	158	956	2278
2013	IPHONE 5 C	26	29942	N.D.	N.D.	1617	N.D.	N.D.	42171	43087	25180	39575	4234	61	27596	176	94	572	N.D.	4638	61	161	6278
2013	GOOGLE Nexus 5	282	37992	101	79	890	N.D.	N.D.	42537	823	29136	5374	18755	44	6763	64	123	N.D.	40	11384	16	764	6369

N.D.: not detected; unit of measurement is mg/kg.

## Results and Discussion

### Metals and hazardous assessment

Metals contained in the 36 WMPs are listed in Table 1. The sum of the 22 metals in the WMPs accounted for 8.94–30.63% of the total, consistent with previous studies^5^. Iron was the most abundant metal (ranging from 2552 to 52765 mg/kg, with an average of 34335 mg/kg), representing about 20% of the total metallic content mainly because of the steel shell. Copper (ranging from 20438 to 37472 mg/kg, with an average of 28351 mg/kg) and aluminium (ranging from 7276 to 62363 mg/kg, with an

average of 27567 mg/kg) ranked next, at similar percentages of about 16% of the total. Copper is primarily used within the printed wiring board (PWB) to facilitate electrical connection between miscellaneous layers in the phone board. Aluminium is mainly present in the batteries of the WMPs as a current collector, PWBs, and shells for lowering weight^23,28^. Chromium and nickel levels ranged from 233 to 77687 mg/kg and 2225 to 54438 mg/kg, with averages of 22112 mg/kg and 16915 mg/kg, and comprised nearly 12.83% and 10% of the total, respectively. Other metals in the ranges of 1–10% were zinc (ranging from 101 to 172783 mg/kg, average of 13319 mg/kg), cobalt (ranging from 47 to 58599 mg/kg, average of 10788 mg/kg), magnesium (ranging from 342 to 21289 mg/kg, average of 9381 mg/kg), tin (ranging from 900 to 11384 mg/kg, average of 5137 mg/kg), and barium (ranging from 432 to 15711 mg/kg, average of 2385 mg/kg), constituting about 7.73%, 6.26%, 5.45%, 2.98%, and 1.38%, respectively. The rest including arsenic, gold, molybdenum, lead, palladium, silver, selenium, thallium, and vanadium, which were at least one order of magnitude lower, and were at levels lower than 1%. Beryllium and cadmium could not be detected in any of the investigated WMPs.

The results of the TCLP tests are presented in Table 2. TCLP leaching concentrations of almost all the tested metals were far below their thresholds, expect for lead of some models which exceeding the threshold of 5 mg·L^−1^. Five of the 36 TCLP lead leaching concentrations, namely, from the NOKIA 7650, MOTO V70, SAMSUNGD508, BLACKBERRY 9900, and IPHONE 5 models, exceeded the limit, at 10.43, 23.78, 19.69, 5.24, and 10.37 mg/L, respectively. Therefore, those five models were classified as hazardous waste.

**Table 2:** Leachates from waste mobile phones according to the Toxicity Characteristics Leaching Procedure (TCLP).

Year of production	Model	Ag	As	Ba	Cd	Cr	Pb
2002	NOKIA 7650	0.061	N.D.	0.962	N.D.	N.D.	**10.430**
2002	MOTO V70	0.031	N.D.	1.472	N.D.	0.014	**23.780**
2003	NOKIA 1100	0.051	N.D.	1.468	N.D.	0.004	0.820
2004	NOKIA 7610	0.047	N.D.	3.003	N.D.	0.024	2.155
2004	MOTO V3	0.058	N.D.	0.924	N.D.	N.D.	0.050
2005	SAMSUNG D508	0.061	0.006	1.480	N.D.	0.005	**19.690**
2005	NOKIA N90	0.046	N.D.	0.576	N.D.	0.028	0.285
2005	SONY ERICSSON K750c	0.053	0.177	1.776	N.D.	0.021	0.224
2006	NOKIA 5200	0.048	0.012	1.901	N.D.	0.020	2.216
2006	SAMSUNG SGH-D908	0.060	N.D.	1.806	N.D.	N.D.	0.175
2006	SONY ERICSSON W700C	0.046	N.D.	1.840	N.D.	0.018	0.182
2006	MOTO A1200	0.025	N.D.	2.855	N.D.	0.022	0.830
2007	IPHONE 1	0.065	N.D.	1.635	N.D.	0.085	0.014
2007	NOKIA N95	0.062	N.D.	2.780	N.D.	N.D.	N.D.
2008	NOKIA E71	0.030	N.D.	1.770	N.D.	0.065	0.170
2008	BLACKBERRY 9000	0.047	N.D.	2.325	N.D.	0.023	0.781
2008	SAMSUNG i908E	0.058	N.D.	0.430	N.D.	0.004	0.065
2008	IPHONE 3 G	0.061	N.D.	1.628	N.D.	0.023	N.D.
2008	GOOGLE G1	0.061	N.D.	1.669	N.D.	N.D.	N.D.
2009	NOKIA N900	0.054	N.D.	1.274	N.D.	0.024	0.043
2009	SAMSUNG S5230	0.061	0.014	1.799	N.D.	N.D.	N.D.
2009	IPHONE 3GS	0.048	N.D.	1.033	N.D.	0.042	1.416
2010	SAMSUNG Galaxy S	0.060	N.D.	1.778	N.D.	N.D.	N.D.
2010	IPHONE 4	0.055	N.D.	0.407	N.D.	0.027	0.630
2011	BLACKBERRY 9900	0.049	N.D.	1.039	N.D.	0.102	**5.239**
2011	IPHONE 4 S	0.055	N.D.	0.375	N.D.	0.028	4.284
2011	SAMSUNG Galaxy Note	0.062	N.D.	0.873	N.D.	N.D.	N.D.
2011	GOOGLE Nexus S	0.060	N.D.	0.728	N.D.	N.D.	N.D.
2012	SAMSUNG Galaxy Note II	0.061	N.D.	1.677	N.D.	N.D.	N.D.
2012	IPHONE 5	0.062	N.D.	0.695	N.D.	0.059	**10.370**
2012	SAMSUNG galaxy nexus	0.061	N.D.	1.191	N.D.	N.D.	N.D.
2012	BLACKBERRY 9850	0.062	0.007	1.875	N.D.	N.D.	0.188
2012	GOOGLE Nexus 4	0.061	N.D.	1.179	N.D.	N.D.	N.D.
2013	SAMSUNG Galaxy Note3 N9000	0.047	N.D.	0.485	N.D.	0.030	0.613
2013	IPHONE 5 C	0.061	N.D.	1.066	N.D.	N.D.	N.D.
2013	GOOGLE Nexus 5	0.061	N.D.	1.187	N.D.	N.D.	N.D.
TCLP limit		5	5	100	1	5	5
Detection limit		0.007	0.053	0.004	0.0027	0.0071	0.042

Note: N.D.: not detected; concentrations in bold are above regulatory limits; unit of measurement is mg/L.

### Potential environmental and human health impact assessment

**Figure 1:**
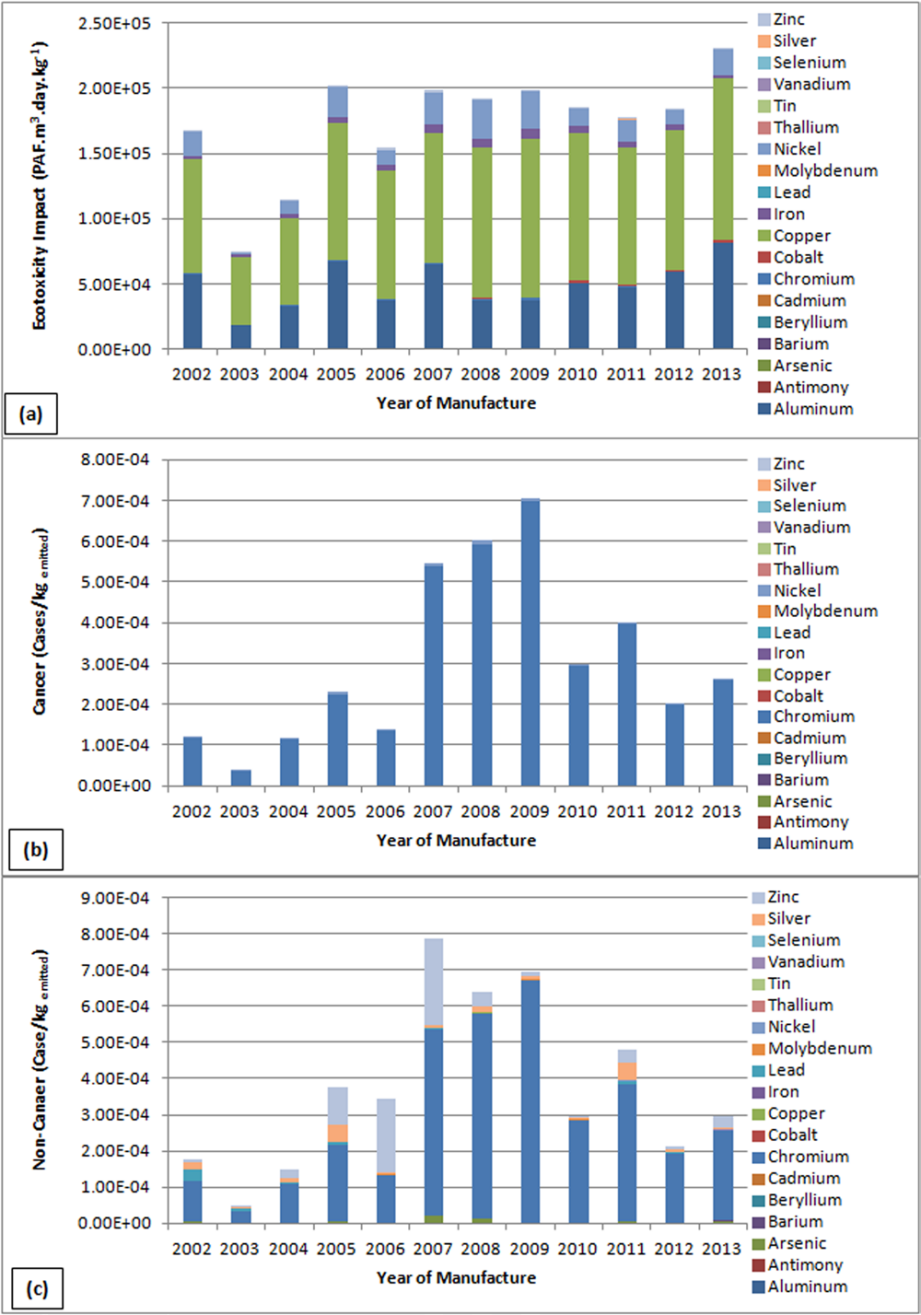
Results of USEtox chemical life cycle assessment of eco-toxicological (**a**), human carcinogenic (**b**), and non- carcinogenic (**c**) impacts of metals in waste mobile phones.

Data obtained from chemical analysis of the cellular phones from 2002–2013 were used with the base data and modelled using USEtox. The results are shown in Fig. 1. Copper posed the most significant ecotoxicity risk (ranging from 52344–123937 PAF·m^3^·day·kg^−1^), followed by aluminium (ranging from 18236–81096 PAF·m^3^·day·kg^−1^), and nickel (10047–30070 PAF·m^3^·day·kg^−1^) which also posed considerable risks. Similar results were also recorded for WPCBs, where copper posed the most significant ecotoxicity risk^7^, ranging from 13273–28153 PAF·m^3^·day·kg^−1^. The two differed in the proportion of copper’s potential ecotoxicity impact, which was about 58% in WMPs but almost 90% in WPCBs. In addition, zinc ranked second for ecotoxicity risk of WPCBs, and the rest were insignificant^7^. Aluminium and nickel ranked second for WMPs as discussed before. This can be attributed to the differences in composition (as shown in Table 1).

Chromium, mainly found in screens, plastics, and shell of alloy steels^21^, exhibited similar tendency for both cancer and non-cancer diseases and showed the most significant risk, ranging from 1.16 × 10^−4^ to 2.57 × 10^−4^ cases/kg_emitted_ and from 1.11 × 10^−4^ to 2.46 × 10^−4^ cases/kg_emitted_, respectively. Chromium for cancer risk weighed almost 98% of the total, and was about 77% for non-cancer risk. The risk potential of zinc (ranging from 1.01 × 10^−5^ to 2.82 × 10^−5^ cases/kg_emitted_), an order of magnitude lower, and silver cannot be neglected. The potential human health risks, both cancer and non-cancer related, are significantly different compared to the results of WPCBs, where lead followed by nickel posed the most significant cancer risk, and zinc followed by lead for non-cancer risks^7^. Similar results were obtained for WMPs by Hilbert and Ogunseitan, where nickel followed by chromium registered the most significant cancer risks; and beryllium followed by lead for non-cancer risks^21^. This can be attributed to the fact that the characterization factors of hexavalent chromium in USEtox Version 2.02 is much higher than that of USEtox Version 1.01, which highlights the potential risk of chromium.

### Technology innovation and regulation development

#### Metals

**Figure 2:**
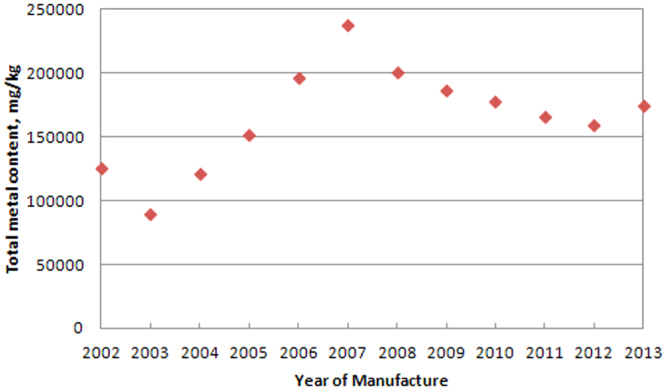
Total metal content in 36 waste mobile phones from 2002 to 2013.

**Figure 3:**
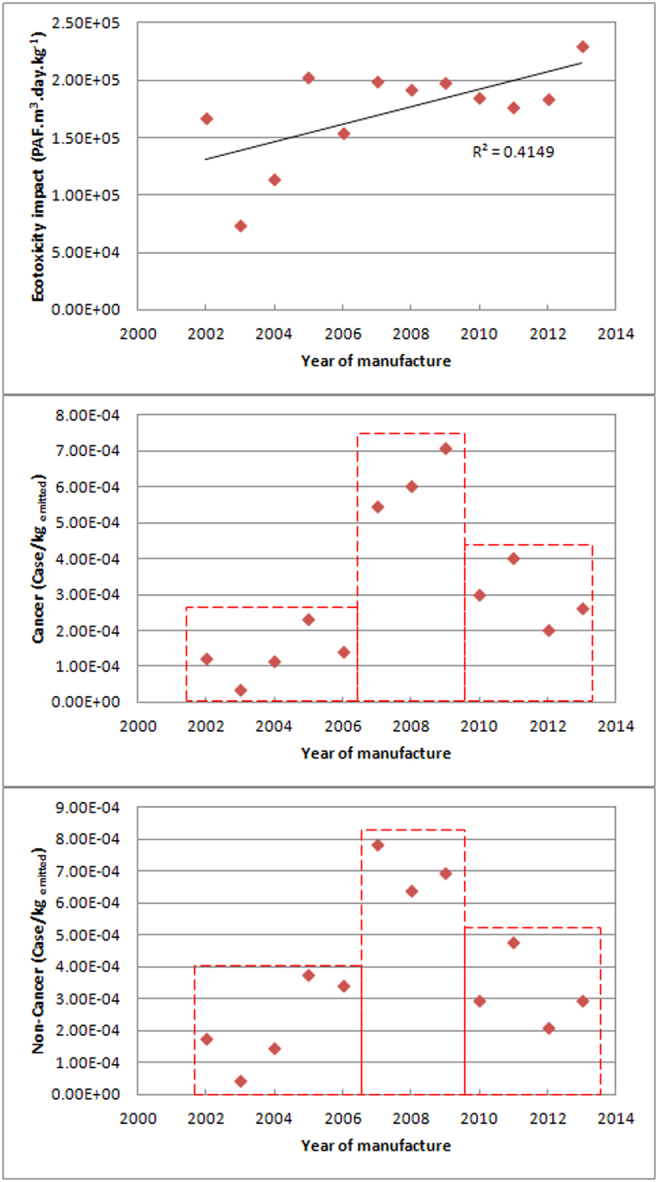
Potential impacts on ecotoxicity and human toxicity (carcinogenic and non- carcinogenic) from 2002 to 2013.

Two milestones, namely, the launch of full touch-screen smart phones in 2007 by APPLE and RoHS implementation by the European Union in 2006, were used to discuss the influence of technology innovation and regulation development on toxicity evolution of WMPs. Figure 2 illustrates integrated metal contents in the WMPs. Figure 3 indicates the potential impact of metals on ecotoxicity and human toxicity.

Figure 2 reveals that the total metal content in the WMPs initially increased from 125,073 in 2002 to 237,316 mg/kg in 2007 and then decreased to 174,745 mg/kg in 2013. It is evident that regulations did not have any notable impact on the total metal content in the WMPs. However, the concentrations of some heavy metals, such as lead (restricted by RoHS in 2006) registered significant decline in 2006, consistent with our previous research^7^ and others^29^. Technology innovation registered a much stronger impact on total metal concentration, which increased from 2002 to 2007 because of functional demands and the uncertainty of future development of mobile phones until the emergence of APPLE’s IPHONE in 2007, which reinvented mobile phones. The impact decreased after 2007 as technology advances after 2007 were used to perfect the blueprint of IPHONEs and limit costs. Therefore, this could guide the production to reduce environmental problem caused by electronic products^30^.

Total metal contained in the Group 2 WMPs (SI Fig. 1) showed the same tendency as Fig. 2, while Group 1 WMPs (SI Fig. 2) appeared to increase from 2002 to 2006 and was stable at around 200,000 mg/kg. After 2006, Group 1 WMPs were mainly from the IPHONE series, and appeared to have two-year cycles: the first year for improvement in technology and the second year for improvement in the software system. Thus, we noted corresponding increase in the total metal concentration in the first year and decrease in the second. Therefore, we concluded that technology innovation had significant impact. Contrarily, regulations barely had an impact.

Technology innovation and regulation development sometimes show associated impacts for specific metals. For example, lead is used as tin-lead solders to attach various components to the PWB in mobile phones. Following its restriction in 2006, and subsequent substitution by silver, silver concentrations in the WMPs should significant increased after 2006^31^. However, we observed that silver concentrations in the WMPs decreased since 2005 (Table 1). This is maybe evidence that other technologies eliminating silver usage for connection were being innovated, reducing the metal content, especially of precious metals^32^. Another example is antimony, which should have increase during the assessment period, as brominated flame retardants were restricted and other flame retardants required Sb_2_O_3_ as an auxiliary fire-resistant agent. However, antimony levels decreased from 623 mg/kg in 2008 to 167 mg/kg in 2013. A possible explanation could be the innovation of environment-friendlier auxiliary fire-resistant agents^33^.

Technology innovation indicated significant impact on single metals. For example, nickel, zinc, molybdenum, iron, and chromium (SI Fig. 3), showed trends that were similar to the total metal contents (Fig. 2) and Group 2 WMPs. Strong evidence could be found in cobalt, magnesium, and vanadium, which increased over the years (SI Fig. 3). Cobalt is the main constituent of batteries, whose numbers increased due to energy demands, especially after the launch of IPHONEs. Magnesium increased from 1302 mg/kg in 2002 to 14157 mg/kg in 2013, which was due to the demand of stylish, portable, and lighter mobiles^34^. This can also explain the slight increase in aluminium, and partly of vanadium, as they are used as alloy metals in steel and in batteries. For WPCBs, an increase in cobalt and vanadium attributable to technology innovation was also indicated by a previous study^7^.

An interesting observation was that the concentrations of some metals or the sum of some precious metals remained at certain values regardless of the advances in technology innovation and regulation development. As shown in SI Fig. 4, copper was around 28,000 mg/kg during 2002–2013. In comparison, copper levels decreased with advancement in technology innovation in WPCBs^7,29^. Some researchers have reported that copper in WMPs was increasing over the years though only samples of 2002, 2005, and 2009 were chosen^35^. Besides, the sum of average concentrations of gold and palladium in WMPs were in the ranges of 80–100 mg/kg regardless of the brands and year of manufacture, as shown in SI Fig. 5. This is interesting, though other reports showed that precious metals decreased because of technology innovation. For example, Chen^7^ reported that technological innovation caused a decline in the use of gold in WPCBs, for cost-effectiveness; Charles^10^ found that the levels of gold was stable from 1991 to 2008, but palladium registered 80% reduction in RAM modules of WEEE.

#### Potential ecotoxicity and human toxicity

The overall trends of potential ecotoxicity and human toxicity displayed diverse increasing trends under the influence of technology innovation and regulations, as shown in Fig. 3. Total ecotoxicity of all the investigated metals increased over the assessment period. Total potential human toxicity of all the investigated metals, for cancer and non-cancer risks, registered a “three step” change: levels in 2002–2006 were at the lowest step, and increased sharply to the highest step in 2007–2009; and finally decreased to the middle step in 2010–2013. This means that the integrated potential toxicity of WMPs increased irrespective of the number of technology innovations and regulations. This result is disappointing as it is very difficult to enact regulations that protect the environment and human health. For example, China took ten years to implement the Chinese WEEE regulation in 2011, though these efforts are not yet to achieve the desired results^20^. This is different to the potential environment and human health impact analysis of WPCBs, where both ecotoxicity and human toxicity showed declining trends with time, indicating that technology innovation and regulation development had postive effects on the environment and human health though the toxicity of some metals such as chromium increased with time^7^. Besides, this implies that the priority of technology innovation is market focus or profitably and not toxicity risk reduction. Technology innovation is a key point in an economic growth engine, meanwhile, economic growth increases the use of technology^36^. Thus, there is an urgent need to balance business profit with environmental benefits^37^.

Toxicity evolution was similar to their corresponding metals concentration as toxicity characterization factors for each metal is specific. Copper was the only exception, as both its ecotoxicity and human toxicity, increased during the assessment period although its concentration remained nearly constant. This is because toxicity is also proportional to metal weight (Equation (1)), and copper weight in the investigated WMPs increased slightly.

## Conclusions

This research demonstrates that WMPs continue to pose considerable threat to ecosystems and public health due to excess toxic metals. Regulation development had positive influence on reducing hazardous risks of a few specific toxic substances such as lead. New materials that are introduced by technology innovation before sufficient assessment exist risks according to our research where ecotoxicity and human toxicity of WMPs increased in the investigated period. This research strongly calls upon the consumers to urge the ICT industry undertake product toxicity risk elimination as their first priority in technology innovation. Additionally, governments at different levels should educate public concerns on sustainability, environment, ecosystem and public health and enable public monitoring the communication industry.

## Electronic supplementary material

Supporting information
