# Supplementary material for: Impact of technological innovation and regulation development on e-waste toxicity: a case study of waste mobile phones
Source: Sci Rep. 2018 May 8;8:7100. doi: 10.1038/s41598-018-25400-0 (PMC5940856; doi:10.1038/s41598-018-25400-0)
Supplement: Supplementary file 1 — Supporting information [file 41598_2018_25400_MOESM1_ESM.docx]

# Supplemental material

Impact of technological innovation and regulation development on e-waste toxicity: a case study of waste mobile phones

**Yu Chen^1^, Mengjun Chen^1^, Yungui Li^1^, Bin Wang^1^, Shu Chen^1^, Zhonghui Xu^1^**

^1^Key Laboratory of Solid Waste Treatment and Resource Recycle (SWUST), Ministry of Education, Southwest University of Science and Technology, 59 Qinglong Road, Mianyang, 621010, China

*Author for correspondence. E-mail: [kyling@swust.edu.cn](mailto:kyling@swust.edu.cn)

Phone: +86-0816-2419569

Contents

**Five figures, three tables are included in this supplemental Material.**

**Fig 1.** Total metal content in waste phones without battery and back shell.

**Fig 2.** Total metal content in waste phones without missing any parts.

**Fig 3.** Nickel, zinc, molybdenum, iron, chromium, cobalt, magnesium and vanadium concentration in waste phones from 2002 to 2013.

**Fig 4.** Copper concentration in 36 waste phones from 2002 to 2013.

**Fig 5.** The sum of gold and palladium concentration in different manufacture.

**Table 1.** Information of 52 waste phones chosen for the study.

**Table 2.** Characteristics of 36 waste phones finally used in the study.

**Table 3.** Classified waste phones into two categories: Group 1 without missing any parts physically, Group 2 without battery and back shell.

**Fig 1.**

**
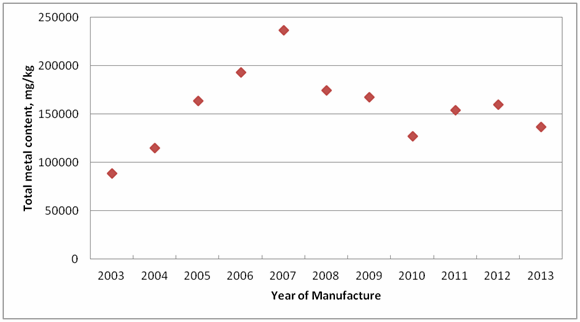
**

**Fig 2.**

**
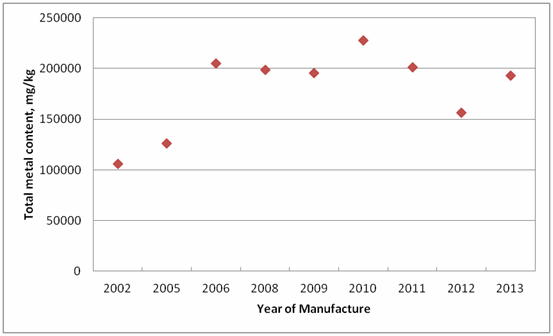
**

**Fig 3.**

**
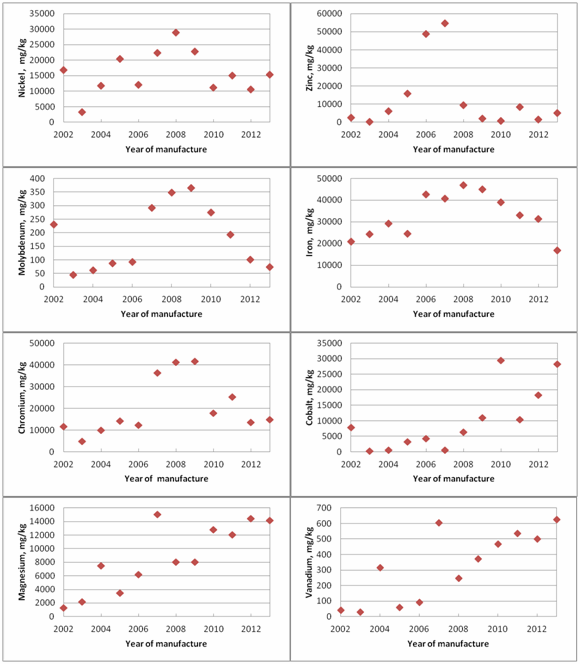
**

**Fig 4.**

**
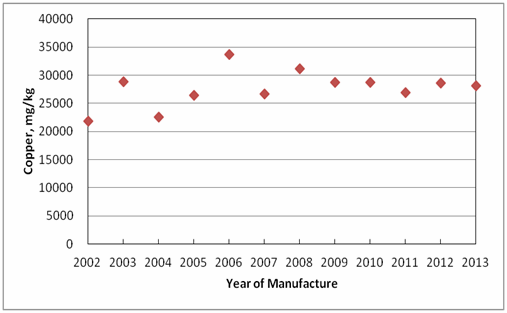
**

**Fig 5.**

**
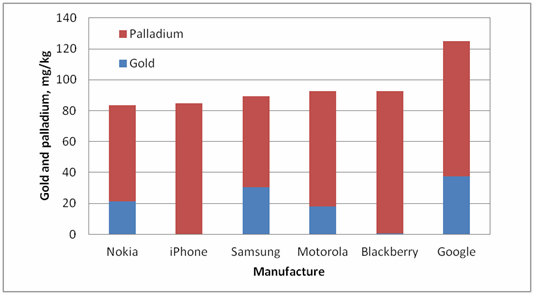
**

**Table 1. Information of 52 waste phones chosen for the study**

| Year | Manufacture | Model |
| --- | --- | --- |
| 2000 | NOKIA | 7110 |
| 2000 | MOTOROLA | A6188 |
| 2000 | SAMSUNG | SGH-M188 |
| 2001 | SAMSUNG | A308 |
| 2001 | NOKIA | 9110 |
| 2001 | MOTOROLA | A6288 |
| 2002 | SAMSUNG | SCH-A599 |
| 2002 | NOKIA | 7650 |
| 2002 | MOTOROLA | V70 |
| 2003 | SAMSUNG | SCH-V208 |
| 2003 | NOKIA | 1100 |
| 2003 | BLACKBERRY | 6230 |
| 2003 | MOTOROLA | A760 |
| 2004 | SAMSUNG | SGH-X608 |
| 2004 | NOKIA | 7610 |
| 2004 | MOTOROLA | V3 |
| 2005 | SAMSUNG | D508 |
| 2005 | NOKIA | N90 |
| 2005 | BLACKBERRY | K750c |
| 2006 | NOKIA | 5200 |
| 2006 | SAMSUNG | SGH-D908 |
| 2006 | BLACKBERRY | W700C |
| 2006 | MOTOROLA | A1200 |
| 2007 | IPHONE | 1 |
| 2007 | NOKIA | N95 |
| 2007 | SAMSUNG | U608 |
| 2007 | MOTOROLA | Q8 |
| 2008 | NOKIA | E71 |
| 2008 | BLACKBERRY | 9000 |
| 2008 | SAMSUNG | i908E |
| 2008 | IPHONE | 3G |
| 2008 | GOOGLE | G1 |
| 2009 | NOKIA | N900 |
| 2009 | SAMSUNG | S5230 |
| 2009 | IPHONE | 3GS 32G |
| 2010 | SAMSUNG | Galaxy S |
| 2010 | IPHONE | 4 |
| 2010 | GOOGLE | Nexus One |
| 2011 | BLACKBERRY | 9900 |
| 2011 | IPHONE | 4S |
| 2011 | SAMSUNG | Galaxy Note |
| 2011 | GOOGLE | Nexus S |
| 2012 | SAMSUNG | Galaxy Note II |
| 2012 | IPHONE | 5 |
| 2012 | SAMSUNG | galaxy nexus |
| 2012 | BLACKBERRY | 9850 |
| 2012 | GOOGLE | Nexus 4 |
| 2013 | SAMSUNG | Galaxy Note3 N9000 |
| 2013 | IPHONE | 5C 16G |
| 2013 | GOOGLE | Nexus 5 |
| 2013 | BLACKBERRY | Z30 |

**Table 2. Characteristics of 36 waste phones finally used in the study**

| **Year** | **Manufacture** | **Model** | **Weight (g)** | **Producing area** |
| --- | --- | --- | --- | --- |
| 2002 | NOKIA | 7650 | 154.30 | CHINA |
| 2002 | MOTOROLA | V70 | 67.33 | AMERIC |
| 2003 | NOKIA | 1100 | 51.76 | CHINA |
| 2004 | NOKIA | 7610 | 88.78 | CHINA |
| 2004 | MOTOROLA | V3 | 80.10 | TAIWAN |
| 2005 | SAMSUNG | D508 | 102.67 | JAPAN |
| 2005 | NOKIA | N90 | 155.46 | FINLAND |
| 2005 | BLACKBERRY | K750c | 78.53 | CHINA |
| 2006 | NOKIA | 5200 | 80.70 | CHINA |
| 2006 | SAMSUNG | SGH-D908 | 81.76 | CHINA |
| 2006 | BLACKBERRY | W700C | 75.52 | CHINA |
| 2006 | MOTOROLA | A1200 | 96.55 | CHINA |
| 2007 | IPHONE | 1 | 113.53 | CHINA |
| 2007 | NOKIA | N95 | 100.66 | CHINA |
| 2008 | NOKIA | E71 | 92.74 | CHINA |
| 2008 | BLACKBERRY | 9000 | 92.16 | HUNGARY 1 |
| 2008 | SAMSUNG | i908E | 81.79 | CHINA |
| 2008 | IPHONE | 3G | 132.86 | CHINA |
| 2008 | GOOGLE | G1 | 137.19 | TAIWAN |
| 2009 | NOKIA | N900 | 152.55 | KOREA |
| 2009 | SAMSUNG | S5230 | 69.52 | CHINA |
| 2009 | IPHONE | 3GS 32G | 134.70 | CHINA |
| 2010 | SAMSUNG | Galaxy S | 75.17 | Unspecfied |
| 2010 | IPHONE | 4 | 137.94 | CHINA |
| 2011 | BLACKBERRY | 9900 | 96.90 | HUNGARY 2 |
| 2011 | IPHONE | 4S | 146.78 | CHINA |
| 2011 | SAMSUNG | Galaxy Note | 116.42 | Unspecfied |
| 2011 | GOOGLE | Nexus S | 88.62 | KOREA |
| 2012 | SAMSUNG | Galaxy Note II | 103.24 | CHINA |
| 2012 | IPHONE | 5 | 112.80 | CHINA |
| 2012 | SAMSUNG | Galaxy nexus | 92.32 | VIETNAM |
| 2012 | BLACKBERRY | 9850 | 92.34 | Unspecfied |
| 2012 | GOOGLE | Nexus 4 | 137.82 | KOREA |
| 2013 | SAMSUNG | Galaxy Note3 N9000 | 112.82 | Unspecfied |
| 2013 | IPHONE | 5C 16G | 129.57 | CHINA |
| 2013 | GOOGLE | Nexus 5 | 136.98 | KOREA |

**Table 3. Classified waste phones into two categories: Group 1 without missing any parts physically, Group 2 without battery and back shell.**

| **Group 1** |  |  |  |
| --- | --- | --- | --- |
| Year | Manufacture | Model | Weight (g) |
| 2002 | NOKIA | 7650 | 154.30 |
| 2005 | SAMSUNG | D508 | 102.67 |
| 2006 | SAMSUNG | SGH-D908 | 81.76 |
| 2008 | IPHONE | 3G | 132.86 |
| 2009 | IPHONE | 3GS 32G | 134.70 |
| 2010 | IPHONE | 4 | 137.94 |
| 2011 | IPHONE | 4S | 146.78 |
| 2012 | IPHONE | 5 | 112.80 |
| 2012 | SAMSUNG | Galaxy nexus | 92.32 |
| 2013 | IPHONE | 5C 16G | 129.57 |
| 2013 | GOOGLE | Nexus 5 | 136.98 |
| **Group 2** |  |  |  |
| Year | Manufacture | Model | Weight (g) |
| 2003 | NOKIA | 1100 | 51.76 |
| 2004 | NOKIA | 7610 | 88.78 |
| 2005 | NOKIA | N90 | 155.46 |
| 2005 | BLACKBERRY | K750c | 78.53 |
| 2006 | NOKIA | 5200 | 80.70 |
| 2006 | BLACKBERRY | W700C | 75.52 |
| 2006 | MOTOROLA | A1200 | 96.55 |
| 2007 | IPHONE | 1 | 113.53 |
| 2007 | NOKIA | N95 | 100.66 |
| 2008 | BLACKBERRY | 9000 | 92.16 |
| 2008 | SAMSUNG | i908E | 81.79 |
| 2009 | SAMSUNG | S5230 | 69.52 |
| 2010 | SAMSUNG | Galaxy S | 75.17 |
| 2011 | BLACKBERRY | 9900 | 96.90 |
| 2011 | SAMSUNG | Galaxy Note | 116.42 |
| 2011 | GOOGLE | Nexus S | 88.62 |
| 2012 | SAMSUNG | Galaxy Note II | 103.24 |
| 2012 | BLACKBERRY | 9850 | 92.34 |
| 2012 | GOOGLE | Nexus 4 | 137.82 |
| 2013 | SAMSUNG | Galaxy Note3 N9000 | 112.82 |
